# Metabolic and Gut Microbiota Responses to Sourdough Pasta Consumption in Overweight and Obese Adults

**DOI:** 10.3389/fnut.2020.615003

**Published:** 2020-12-23

**Authors:** Shrushti Shah, Paul D. S. Brown, Shyamchand Mayengbam, Michael G. Gänzle, Weilan Wang, Chunlong Mu, Silvio Lettrari, Craig Bertagnolli, Jane Shearer

**Affiliations:** ^1^Faculty of Kinesiology, University of Calgary, Calgary, AB, Canada; ^2^Department of Biochemistry, Memorial University of Newfoundland, St. John's, NL, Canada; ^3^Department of Agricultural, Food and Nutritional Science, University of Alberta, Edmonton, AB, Canada; ^4^Kaslo Sourdough, Kaslo, BC, Canada; ^5^Department of Biochemistry and Molecular Biology, Cumming School of Medicine, University of Calgary, Calgary, AB, Canada

**Keywords:** sourdough pasta, obesity, microbiota (microorganism), glycemic index, fermenation

## Abstract

Increasing consumer interest in fermented products has driven the emergence of a number of novel foods including shelf-stable sourdough pasta. This study comprehensively examined the impact of fermentation on the microbial composition of the culture, pasta, its subsequent effects on glycemic responses and gut microbiota in overweight men and women (>25 kg/m^2^) compared to a conventional, non-fermented pasta. Two, randomized crossover trials were performed. Study A examined acute feeding responses to each product wherein fasted participants completed a meal tolerance test comprised of 75 g of conventional or sourdough pasta to examine glycemic responses. Results showed enhanced gastric emptying with sourdough, but no difference in overall blood glucose, insulin or satiety hormone responses between the treatments. Study B consisted of three standard oral glucose tolerance tests as well as fecal collection for sequencing at baseline and following each pasta intervention (150 g or 2 serving/d for 5 days) followed by a 2-week washout period. Results showed no differential impact of either pasta treatment on glucose tolerance. Analysis of fecal bacterial and fungal (mycobiome) microbiota showed no change at the individual species or genus levels. However, fungi were adaptive following chronic pasta consumption with decreases in alpha diversity of fungi following sourdough, but not conventional pasta. This was accompanied by reductions in total fecal short chain fatty acid concentrations. In conclusion, sourdough fermentation did not change the overall glycemic properties of the pasta, incretin responses or bacterial gut microbiota, but appears to impact microbiome fungal community structure with chronic consumption.

## Introduction

Foods derived from cereal grains, such as bread and pasta are key constituents of the diet and represent staple foods in many countries. Lowering the glycemic index of staple foods is of great interest as such foods are generally consumed in large quantities, have high caloric density and are implicated in the rising obesity epidemic (1). Furthermore, there is growing consumer interest in foods with improved nutritional and functional properties.

Sourdough fermentation is an ancient technology traditionally used to leaven bread (2). Sourdough cultures are typically dominated by lactic acid bacteria in symbiotic combination with yeasts (3, 4). Lactic acid bacteria are known to make species- or strain specific contributions to product quality but sourdough fermentation generally increases acidity through production of lactic and acetic acids (3, 5). Sourdough fermentation increases protein digestibility, total soluble fiber content, reduces the glycemic index of food and improves the bioavailability of minerals (6, 7). In addition, fermentation is considered to be a sustainable and effective method to make whole grain and fiber-rich products more palatable while also improving the functional and nutritional value of the products (8).

While traditionally used in bread making, sourdough cultures are becoming more prominent in other novel foods including the use of sourdough in pasta making (6, 9). Conventional pasta typically has a high glycemic index, wherein frequent exposure can have a negative impact on glucose tolerance (1). Given this, the purpose of the present study was to examine the impact of fermented sourdough pasta on postprandial glycemic responses, gut hormones, gastric emptying, gut microbial diversity and fecal short chain fatty acids (SCFA) compared to conventional non-fermented pasta in overweight and obese adults. We hypothesized that sourdough pasta would decrease postprandial blood glucose responses and also result in differential gut microbiome profiles compared to conventional pasta consumption.

## Methods

### Participants and Study Design

**Figure 1:**
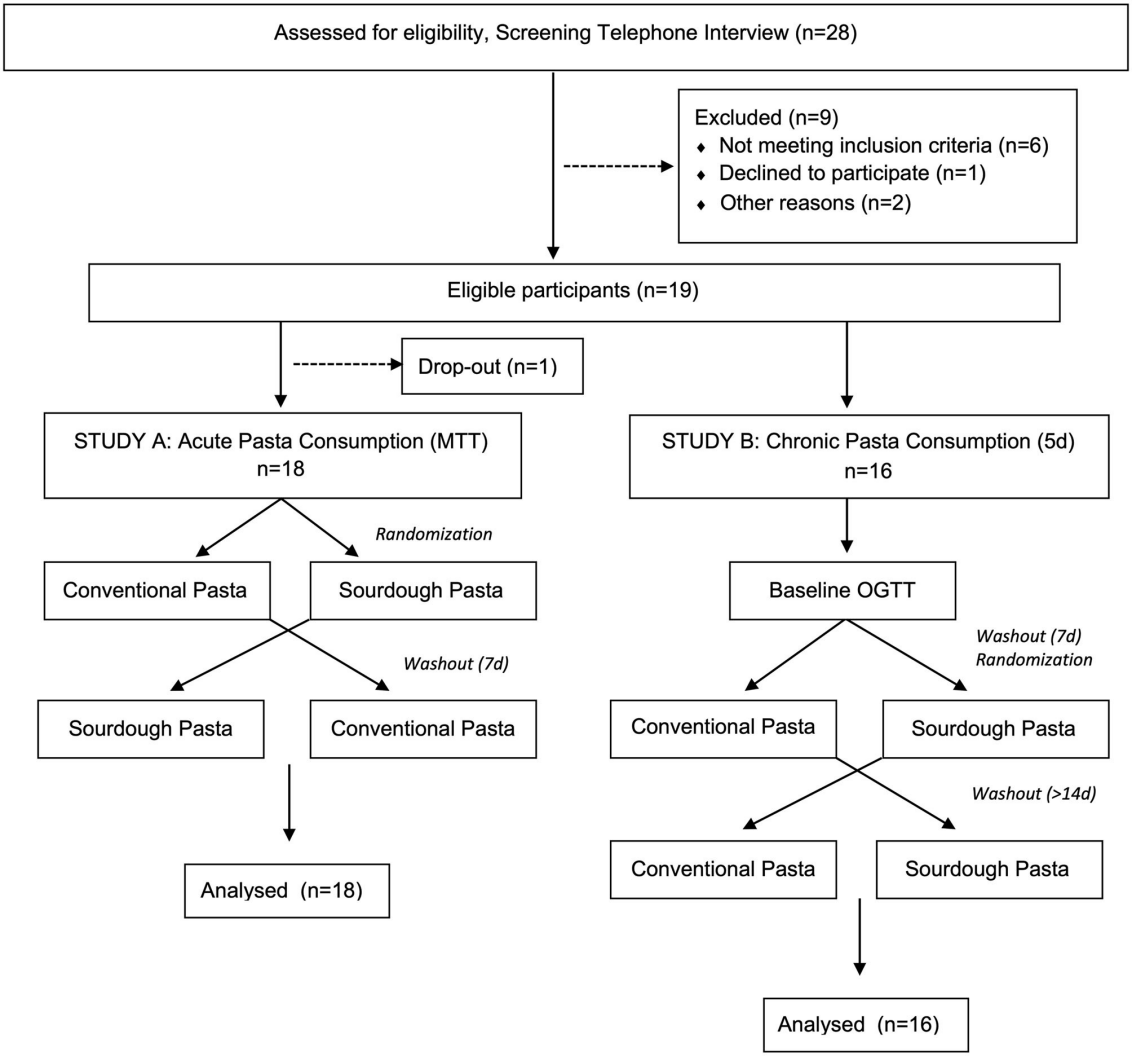
CONSORT flow chart of participants enrolled in the study. In study A, 18 volunteers were randomly assigned into meal tolerance tests (MTT) consisting of either conventional or sourdough pasta in a cross-over design. Following study A, 16 of the same participants volunteered for study B where they underwent a baseline oral glucose tolerance test (OGTT) followed by randomization into either conventional or sourdough pasta consumption for 5 days.

This randomized, double-blind, crossover design study was approved by the Conjoint Health Research Ethics Board at the University of Calgary (REB16-2317). Participant inclusion criteria included >25 years of age and a body mass index (BMI) > 25 kg/m^2^ at the time of the study. Researchers initially screened volunteers for overall health status and excluded those with a medical history of gluten allergy, dyslipidemia, gastrointestinal disease, diabetes, other chronic diseases, as well as those taking prescription medications, antibiotics (past 6 months) or natural health products (e.g., already consuming regular pre- or probiotics). Pregnant volunteers and smokers were also excluded from participating. A CONSORT flow chart of participants enrolled in the study is shown in Figure 1. Following informed written consent from eligible volunteers, basic demographic information (e.g., age, sex), anthropometric measures (e.g., height, weight, calculated BMI, waist circumference, hip circumference), and health measures (e.g., resting blood pressure, resting heart rate) were collected. A full-body dual-energy x-ray absorptiometry (DEXA) scan was performed on each participant to analyze body composition. Self-report questionnaires were administered to participants to gather information regarding family medical history.

### Sourdough Culture

The sourdough culture is maintained by back-slopping at Kaslo Sourdough (Kaslo, BC, Canada) for the purpose of making bread and pasta. To characterize the microbial composition of the sourdough culture, bacterial and fungal composition was assessed at two time points. The first (SD1) was the starter (mother) culture, while the second renewed (daughter) culture had added flour and water and was mixed before fermentation at ambient temperature for 14–18 h (SD2).

### Conventional and Sourdough Pasta

Conventional (Catelli® Classic Rotini, Catelli Food Corporation. Winnipeg, MB, Canada) and sourdough pasta (Kaslo Sourdough Pasta Fermentata™ Classic Rotini, Kaslo BC, Canada) were tested in the present study. Both are shelf stable, dried pasta products that are widely available in Canada. The nutritional profile of each pasta is shown in Table 1. Pastas were compared on a per mass basis (grams) to simulate equivalent consumer consumption. Although not exact in terms of their composition, differences in overall macronutrient composition are minimal. For all studies, pasta was cooked *al dente* according to manufacturers' instructions. Visually, there was no overt difference between the two pasta products with both having the same color, texture and classic rotini shape.

**Table 1:** Macronutrient composition of the pasta per 75 g serving.

	**Conventional dried pasta**	**Sourdough dried pasta**
Grams (g)	75.0	75.0
Calories (kcal)	282.4	262.5
Fat (g)	1.3	0.9
Cholesterol (mg)	0.0	0.0
Sodium (mg)	0.0	0.0
Carbohydrate (g)	56.5	52.5
Fiber (g)	2.6	2.8
Sugars (g)	2.6	0.9
Protein (g)	9.7	8.4
Vitamin A (%)	0.0	0.0
Vitamin C (%)	0.0	0.0
Calcium (%)	1.9	0.0
Iron (%)	9.4	20.0

*% represents percent daily value*.

### Study A: Acute Pasta Consumption

Study A investigated gastric emptying, blood glucose and gut peptide responses following pasta consumption. Eligible participants were asked to come into the laboratory to complete two meal tolerance tests. On the day of the appointment, participants arrived at the laboratory following an overnight fast (10–12 h), abstaining from alcohol, caffeine, strenuous physical activity, and analgesic medications for 24–48 h prior. Once screened, an indwelling catheter was placed in the antecubital vein by a trained nurse to facilitate repeated blood sampling. After baseline blood collection (t = 0 min), participants consumed either 75 g dry weight of conventional or sourdough pasta in a randomized order along with 1 g of paracetamol to track gastric emptying as described (10, 11). All pasta was served plain and consumed within 6 min. Additional blood samples were collected at 30, 45, 60, 75, 90, 105, 120, 150, and 180 min. A minimum 7-day washout period occurred before the entire procedure was replicated with the other treatment.

### Study B: Chronic Pasta Consumption

Study B investigated the impact of chronic pasta consumption over the course of 5 days. This protocol consisted of three standard oral glucose tolerance tests (OGTT) as well as fecal collection at baseline and following each pasta intervention. All participants provided a baseline fecal sample and completed an OGTT (75 g Trutol) following the pre-study procedures outlined in Study A. Blood samples were collected at baseline and at 15, 30, 60, 90, and 120 min. Following baseline sampling, subjects consumed 150 g dry weight/d of either sourdough or conventional pasta for five consecutive days followed by another OGTT (morning of Day 6) and provided an additional fecal sample. After a washout period of at least 2 weeks, but not more than 4 weeks, participants then switched to the other treatment and repeated the protocol.

### Blood Analyses

At each time point, approximately 5 mL of venous blood was collected. Samples were aliquoted into a 2 mL into a K2EDTA plasma vacutainer, 2 mL into a serum vacutainer, and 1 mL into an Eppendorf tube containing EDTA, diprotinin-A (0.034 g/L blood; MP Biomedicals), sigma protease inhibitor (1 g/L blood; Sigma Aldrich), and Roche Pefabloc® SC (1 g/L of blood, Sigma Aldrich) to preserve analytes. Tubes were centrifuged for 15 min at 4°C. Supernatant was removed from each sample and transferred into a new labeled Eppendorf tube for storage at −80°C until analysis. Blood samples collected in Study A were assessed by a custom Human Metabolic Array [C-peptide, glucagon, gastric inhibitory peptide (GIP), glucagon-like peptide-1 (GLP-1 active), insulin and peptide YY (total)] (Milliplex Multiplex, Eve Technologies, University of Calgary). A glucose oxidase assay kit used plasma samples to measure plasma glucose concentrations at all time points (Sigma Aldrich). Serum paracetamol concentrations, determined using a paracetamol assay kit (Cambridge Life Science), were used to track gastric emptying. Several studies have demonstrated paracetamol concentrations to be a valid measure of gastric emptying (10, 11).

### Pasta Microbial Analyses

Under sterile conditions 2.2 g of cooked pasta was homogenized into 5 mL of purified, distilled laboratory grade water for analysis. For bacterial and fungal sequencing, DNA was then extracted as per the below described protocol. To assess total bacterial and fungi in the pastas, PCR was performed as previously described (12) using the following primers for bacterial 16S rRNA genes (Forward: TGAAGAATTGATGGAACTCG, Reverse: CATTGTGGTTCACCGTTC, Tm = 63°C) or the internal transcribed spacer region 1 (ITS1) of fungi (Forward GAGGAAGTAAAAGTCGTAACAAGGTTTC, Reverse GTATCGCATTTCGCTGCGTT, Tm = 56°C). Standard curves were normalized to the copy number of 16SrRNA genes and ITS1 region using reference strain genome size and gene copy number values obtained from the following reference (http://cels.uri.edu/gsc/cndna.html) for both, bacteria and fungi groups. Data are expressed as gene copies/g pasta water slurry described above. It should be recognized that bacteria and fungi in each pasta are not viable, and that the above analysis was done to highlight differences between the two pastas resulting from the fermentation process. Sourdough samples SD1 and SD2 as well as conventional and sourdough pasta were additionally characterized by high throughput sequencing of 16S rRNA and ITS2 sequence tags as outlined below.

### Fecal Microbial Analyses

Following collection, fecal samples were stored at −80°C. DNA was extracted by a PowerMag Soil DNA Isolation kit (MP Biomedical) as per protocol. 16S rRNA genes and ITS2 regions were targeted for bacterial and fungal sequencing, respectively. PCR amplification was done with dual-barcoded primers targeting the V4 region and sequenced with an IIlumina MiSeq using the 300-bp paired-end kit. Primers and PCR conditions used for 16S sequencing were followed as described (13); those used for ITS2 sequencing were identical to Gweon et al. (14). Sequences were denoised, taxonomically classified using Greengenes (v. 13_8) as the reference database, and clustered into 97%-similarity operational taxonomic units (OTUs) with the mothur (15), following the recommended procedure (https://www.mothur.org/wiki/MiSeq_SOP; accessed Nov 2017). Paired-end reads were merged and curated to reduce sequencing error. The processing pipeline for investigating fungal populations was identical to the one used for bacteria, except for the following differences: (1) paired-end reads were trimmed at the non-overlapping ends, and (2) high quality reads were classified using UNITE (v. 7.1) as the reference database. The resulting dataset had 23008 OTUs (including those occurring once with a count of 1, or singletons). An average of 28163 quality-filtered reads were generated per sample. Sequencing quality for R1 and R2 was determined using FastQC 0.11.5. A consensus taxonomy for each OTU was obtained and the OTU abundances were then aggregated into genera with modifications, accounting for the current taxonomy of lactobacilli (16). The datasets generated for this study can be found in NCBI BioProject, NCBI Accession No. PRJNA668386.

### Fecal Short Chain Fatty Acid Analyses

For SCFA of fecal samples, methods followed those previously described (17). Briefly, 200 mg of sample was weighed and internal standard (2-ethylbutyric acid) was added. Samples were dissolved in 95% ethanol and homogenized for two cycles. The samples were stored overnight in −80°C and SCFA were derivatized to its hydrazide forms for further analysis using High Performance Liquid Chromatography (HPLC) as described previously with some modifications (18). An aliquot of 20 μL of sample was injected into the HPLC. The analytes were separated with the use of a C18 column (150 mm × 4.6 and 2.6 um pore size) at 40°C temperature. A gradient method was applied by using acetonitrile and water as solvents at a flowrate of 0.8 mL/min. SCFA were detected at a wavelength of 230 nm and corresponding peaks were integrated using the OpenLab software from Agilent Technologies.

### Statistical Analysis

Participant data are presented as mean ± SD. All the other data are presented as mean ± SEM unless otherwise indicated. Normality of the data was assessed using the Shapiro-Wilk normality test. Differences between treatments were determined using two-way repeated measures ANOVA and Tukey's multiple comparison *post-hoc* test for repeated measures for timed data. Area under the curve (AUC) was calculated using the trapezoid method (19). Statistical analysis was performed using GraphPad Prism (v. 7.0; La Jolla, USA) and the mothur software package (15). Alpha diversity for bacterial and fungal reports was estimated with the Shannon index on raw OTU abundance tables after filtering out contaminants. The significance of diversity differences was tested with an ANOVA. To estimate beta diversity across samples, we excluded OTUs occurring with a count of less than three in at least 10% of the samples and then abundance-weighted sample pair-wise differences were computed using Bray-Curtis dissimilarity. Beta diversity was visualized using Principal Coordinates Analysis (PCoA) ordination. Variation in community structure was assessed with permutational multivariate analyses of variance (PERMANOVA) with treatment group as the main fixed factor and using 4,999 permutations for significance testing. All analyses were conducted in the R environment. Statistical significance was set at *p* < 0.05.

## Results

### Culture-Independent Analyses of Sourdoughs

**Figure 2:**
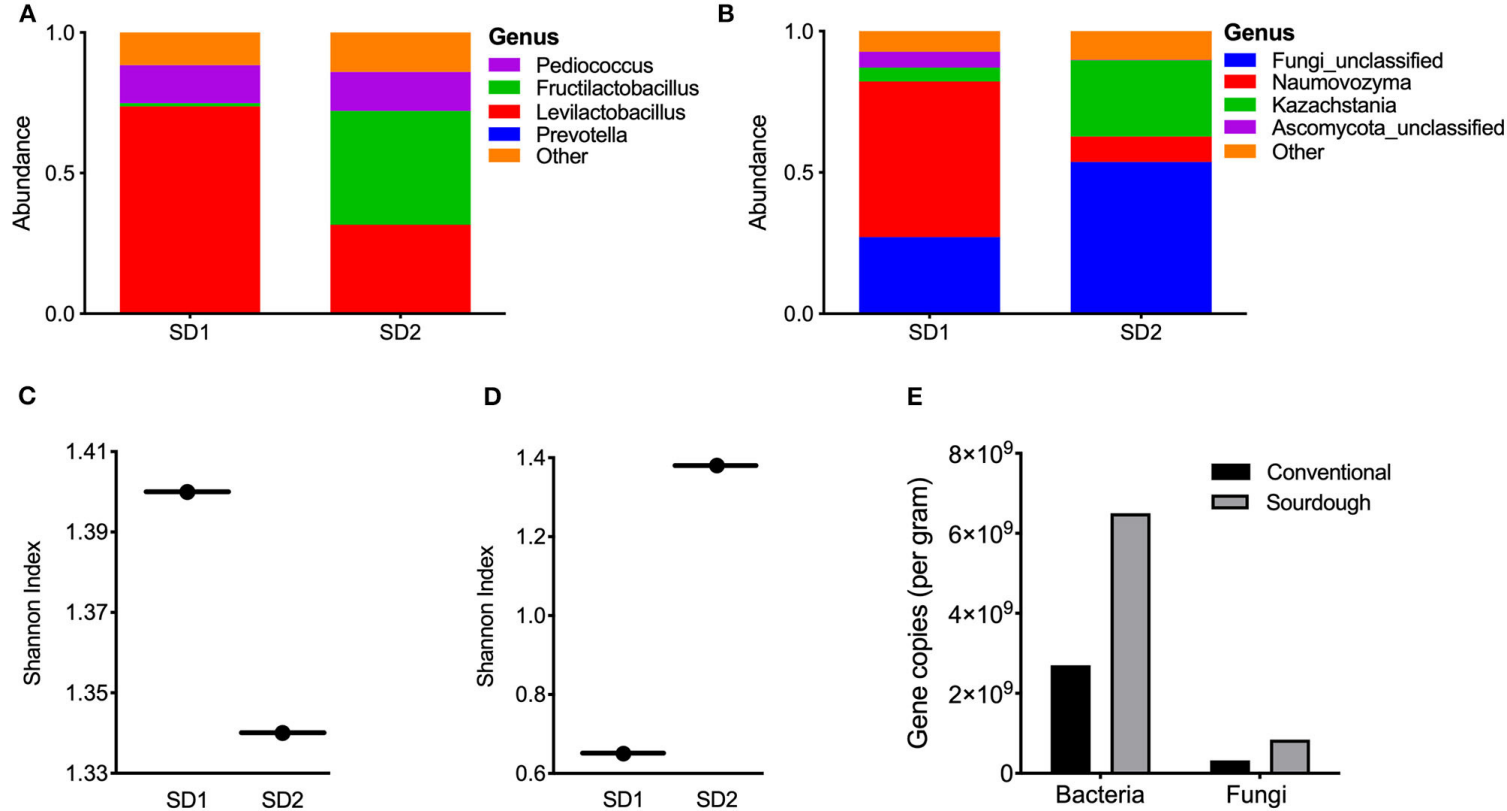
Bacterial and fungal composition of sourdough culture at day 0 (mother culture, SD1) and day 1 (daughter culture, SD2). **(A)** Bacterial genus abundance and **(B)** alpha diversity in SD1 and SD2 for bacteria in cultures, **(C)** Fungal genus abundance, **(D)** alpha diversity in SD1 and SD2 for fungi in cultures. The starter (mother) culture was exposed to air for 14–18 h after addition of flour, water and mixing to make daughter culture (SD2). **(E)** Results of PCR for bacterial and fungi abundance in pasta samples, expressed per gram of sample (see Methods).

Sourdough cultures are extremely complex, and vary in their composition. They can contain two to five of more than 100 different species of lactic acid bacteria and one to three of more than two dozen species of yeasts. For this reason, the microbial composition of the culture used in this study is reported. To determine the microbial composition of the sourdough culture, sequencing was performed. Results are reported at the genus level (Figure 2). The mother culture (SD1) was dominated by *Pediococcus* (15%) and *Levilactobacillus* (74%); most sequences matched most closely to *Levilactobacillus parabrevis* (Figure 2A). In the second sourdough stage after 14–18 h of fermentation at ambient temperature (SD2), the abundance of *Pediococcus* remained unchanged, *Levilactobacillus* were reduced to 40% and sequences matching the genus *Fructilactobacillus* increased (41%). There was bacterial alpha diversity in SD1 and SD2 remained essentially unchanged (Figure 2B). Fungal analysis showed the SD1 culture to be dominated by *Naumovozyma* genus with *Naumovozyma castelli* (anamorph: *Saccharomyces castelii*) accounting for 55% of abundance. Levels of the *Naumovozyma* genus were reduced in SD2 with growth of *Kazachstania barneti* (26.9%) and other unclassified fungi (53.7%) (Figure 2C). Overall, fungal alpha diversity increased in SD2 (Figure 2D). Additional data relating to culture composition can be found in Supplementary Figure 1.

### Pasta Analyses

To further distinguish the two pasta products and highlight microbial changes taking place with fermentation, the microbial composition of each pasta was assessed. However, it must be realized that the microbes in each were no longer viable. Results of bacterial and fungal profiles are shown in Supplementary Figures 2, 3. Conventional pasta was dominated by *Prevotella* (15%) while sequences representing the genera *Pediococcus* (64%) and *Levilactobacillus* (28%) were the most prevalent species in the sourdough pasta. Together, these two genera accounted for 95% of the sequences from sourdough pasta. Relative to bacteria, both pasta products displayed minimal fungal diversity with most being unclassified. Analysis of total bacterial and fungal content in the samples showed the sourdough pasta to contain greater counts of bacteria and fungi compared to the conventional product (Figure 2E).

### Participant Characteristics

Characteristics of participants are shown in Table 2. Study A consisted of 18 participants while Study B consisted of 16 of the same participants. The age and cardiometabolic parameters (BMI, Waist/Hip circumference, resting blood pressure, heart rate) and DEXA scan results were also similar in both, Study A and Study B participants (*p* > 0.05).

**Table 2:** Baseline characteristics of participants.

	**Study A**	**Study B**
**Total participants, n**	18	16
Age (years)	38.6 ± 10.6	42 ± 10
Males/females	6/11	5/11
BMI, kg/m^2^	29.6 ± 1.0	28.46 ± 2.8
Waist circumference, cm	95.7 ± 3.1	93.71 ± 9.1
Hip circumference, cm	108.9 ± 2.1	106.13 ± 7.2
Systolic BP, mmHg	124.5 ± 3.7	117.87 ± 14.9
Diastolic BP, mmHg	72.3 ± 1.9	68.37 ± 9.3
Resting HR, bpm	68.9 ± 2.2	63.93 ± 8.3
Total BMD, g/cm^2^	1.14 ± 0.0	1.16 ± 0.1
% Fat arm	34.5 ± 9.9	31.60 ± 10.8
% Fat trunk	32.4 ± 7.6	28.21 ± 8.4
% Fat leg	33.0 ± 11.2	31.69 ± 9.9
Total % fat	32.1 ± 2.0	29.29 ± 8.2

*Values represent as mean ± SD. BMD, bone mineral density; BMI, body mass index; BP, blood pressure; HR, heart rate*.

### Study A. Acute Pasta Consumption

**Figure 3:**
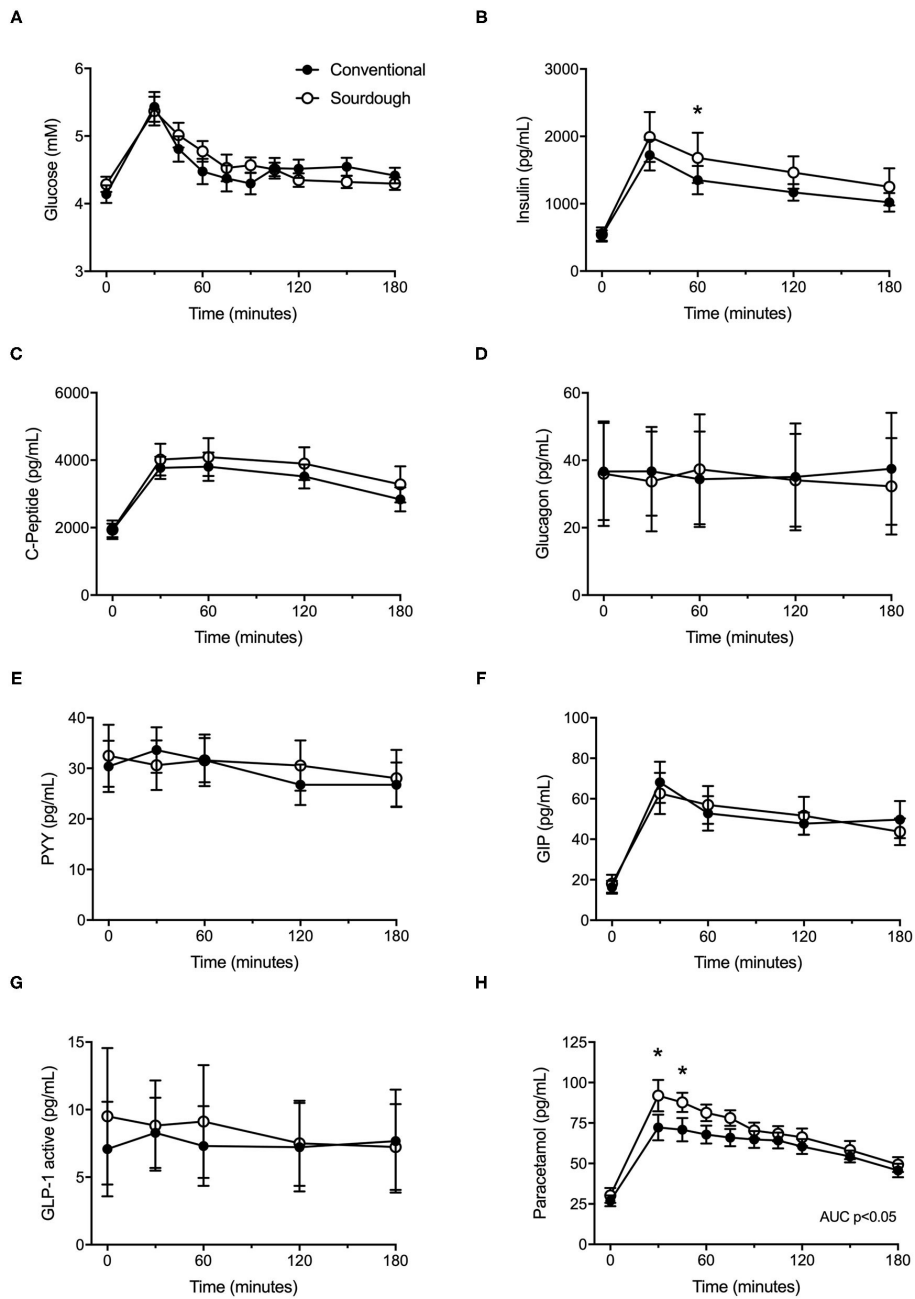
Effect of acute intake of conventional and sourdough pasta on, **(A)** blood glucose, **(B)** insulin, **(C)** C-peptide, **(D)** Glucagon, **(E)** PYY, **(F)** GLP-1, **(F)** GIP and **(H)** paracetamol. All data are presented as mean ± SEM (*n* = 18). Abbreviations: GIP – gastric inhibitory polypeptide, GLP1 – glucagon-like peptide, PYY – peptide YY. *Indicates statistical significance (*p* < 0.05) compared to conventional pasta treatment at a corresponding time point.

To assess the impact of acute pasta consumption, each participant consumed 75 g of conventional or sourdough pasta, followed by assessment of their glycemic response. No differences in baseline glucose or insulin levels were present prior to meal tolerance tests between conventional or sourdough pasta administration (*p* > 0.05). Following pasta administration, there were no significant differences in the overall responses (total AUC) of glucose (Figure 3A, *p* > 0.05) and insulin (Figure 3B, *p* > 0.05). However, insulin levels were greater with sourdough at 60 min post-consumption (*p* < 0.05). No differences between conventional or sourdough pasta consumption for gut peptide responses including C-Peptide, glucagon, PYY, GIP or GLP-1 were found (Figures 3C–G, *p* > 0.05). The total serum paracetamol levels (AUC), a proxy measure of gastric emptying was elevated with sourdough consumption (Figure 3H, *p* < 0.05).

### Study B. Chronic Pasta Consumption

**Figure 4:**
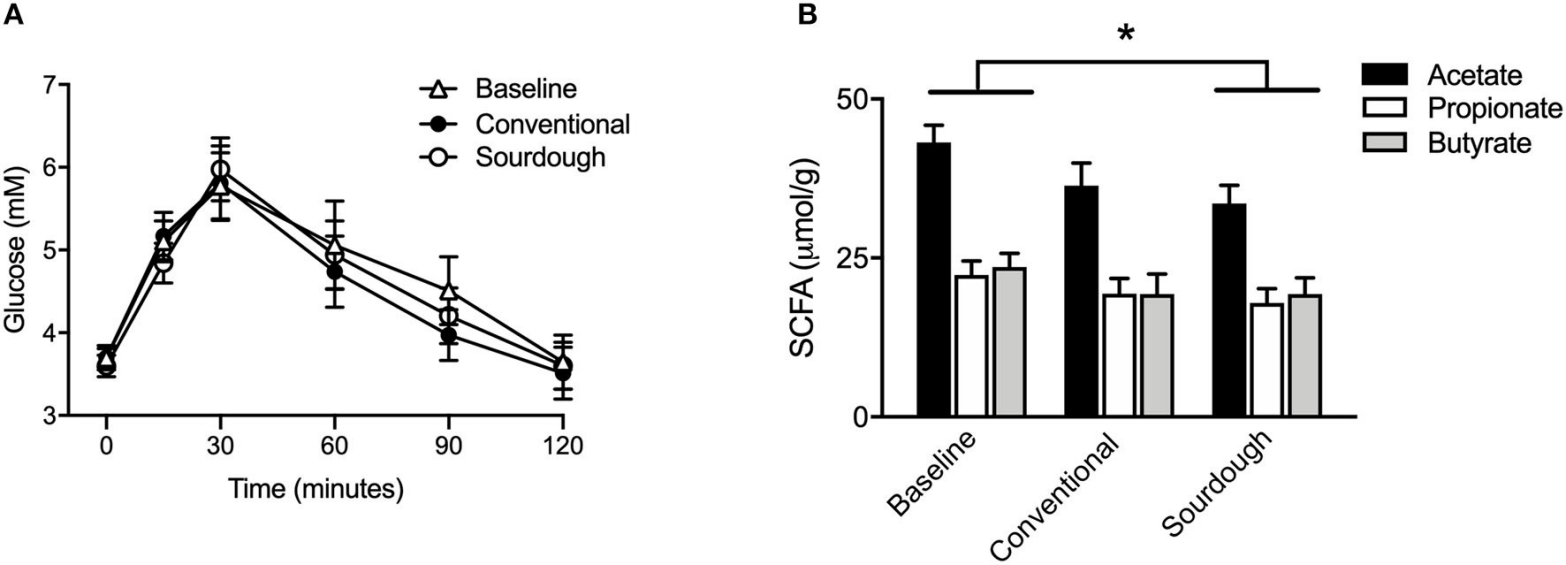
Effect of chronic intake of conventional and fermented sourdough pasta on **(A)** blood glucose, **(B)** fecal SCFA concentration. All data are presented as mean ± SEM (*n* = 16). *Indicates statistical significance in total SCFA concentration (*p* < 0.05) compared to baseline values.

To assess the impact of chronic pasta consumption, subjects completed a baseline OGTT followed by 5 days of pasta consumption (150 g/day) in a cross-over design. Similar to previous study A, there was no difference in blood glucose levels prior to OGTT administration in any trial. Likewise, there were no differences in glucose or insulin excursion between baseline (no pasta), conventional and sourdough pasta (Figure 4A, *p* > 0.05). As the benefits of fermented foods may involve the gut microbiota, microbial metabolites including SCFA (acetate, propionate, butyrate) were analyzed in fecal samples. There was no difference in the concentration of individual fecal SCFA at baseline or with each pasta treatment (Figure 4B, *p* > 0.05). However, the concentration of total SCFA concentration decreased in the sourdough treatment compared to baseline values (*p* < 0.05).

**Figure 5:**
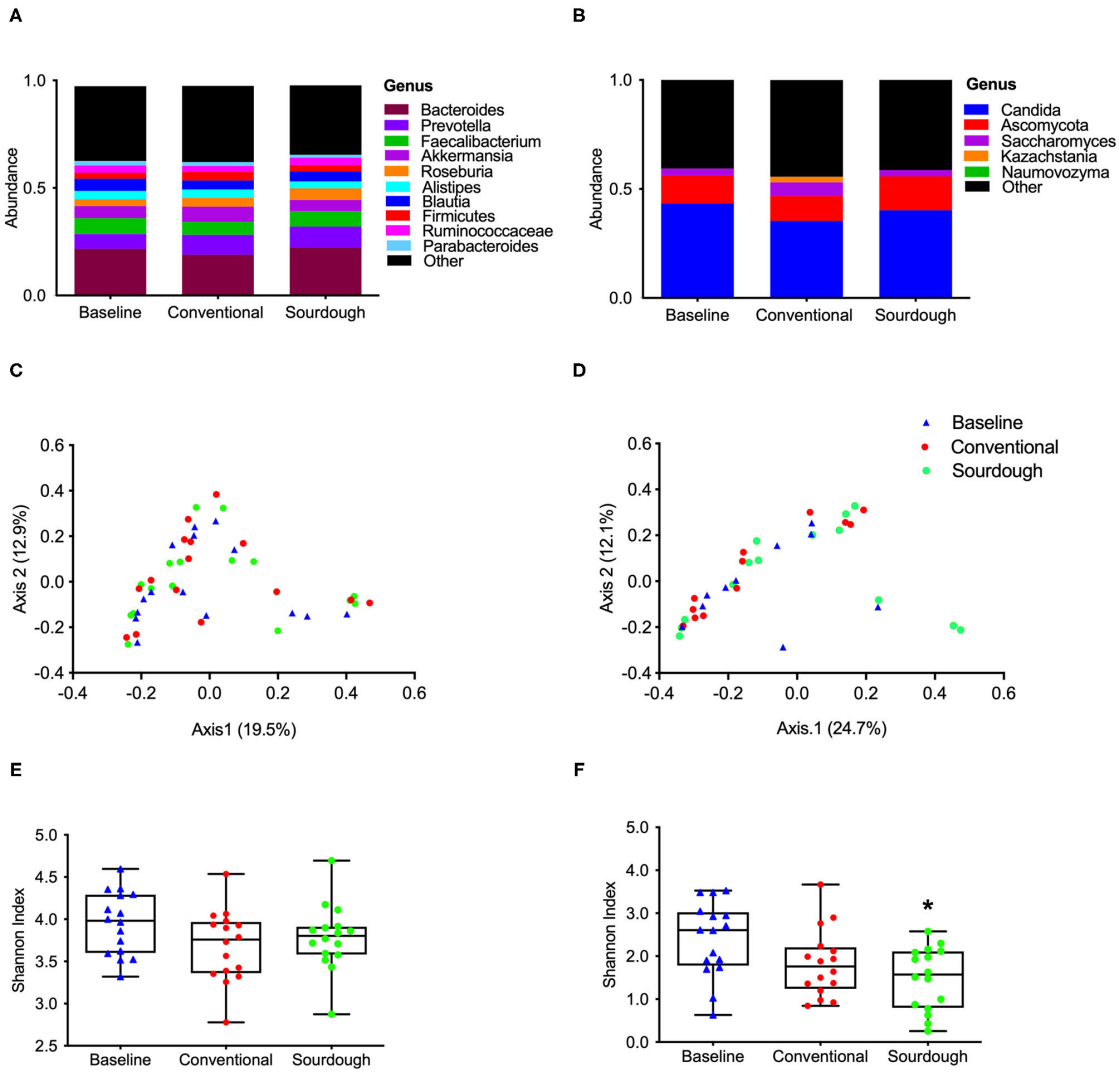
Impact of chronic consumption of conventional vs. sourdough pasta on bacterial and fungal diversity of overweight participants after consumption of conventional and sourdough pasta for 5 days. **(A)** Bacterial and **(B)** fungal abundance; **(C)** bacterial and **(D)** fungal beta diversity; **(E)** bacterial and **(F)** fungal alpha diversity assessed using Shannon Index. *Indicates statistical significance (*p* < 0.05) compared to baseline values.

To investigate the potential impact of pasta consumption on the gut microbiota, both 16S and ITS2 sequencing were performed. No treatment differences were observed for bacterial (Figure 5A) and fungi (Figure 5B) at the phyla, class, family or genus levels (*p* > 0.05, Supplementary Figures 4, 5). We next assessed the alpha and beta-diversity as a reflection of how each pasta treatment changed microbial structure (Figures 5C–F). There were no changes found in alpha diversity (*F* = 1.88, *P* = 0.17) as assessed by the Shannon Index (Figure 5C, *p* > 0.05) of bacterial groups with treatment; however, there was a significant decrease in alpha diversity in fungi (*F* = 5.37, *P* = 0.008) with chronic consumption of sourdough (Figure 5D, *p* < 0.05). These differences in microbial structure are also reflected in the separation between groups when plotted as principal coordinates (Figures 5E,F).

## Discussion

Sourdough fermentation has the potential to increase nutrient bioavailability, decrease the glycemic index and gluten content of foods along with improving product shelf life (20). In the present study, we examined the impact of fermentation on the microbial composition of pasta as well as the acute and chronic effects of consumption on glucose regulation and gut microbial composition in overweight and obese adults. Major findings indicate that acute or chronic consumption of sourdough pasta had no effect on glycemia and other gut hormones in participants compared to conventional, non-fermented pasta. Of interest, we found that the microbiome was adaptive following chronic pasta consumption with reductions in fecal SCFA and decreases in the alpha diversity of fungi following sourdough, but not conventional pasta.

The approaches employed in this study are unique in that we tracked the fungal and bacterial composition from the starter culture to the pasta and finally its subsequent impact on the human microbiota. In addition, this is the first report on the microbiota of a sourdough that is maintained for commercial pasta production. Culture-independent analyses of the mother dough (SD1) showed a dominance of *Levilactobacillus* species that diversified upon maturation in the second stage of sourdough fermentation (SD2), where *Pediococcus* species and *Fructilactobacillus* species were additionally present in high numbers. More than 50 microbial species of lactic acid bacteria including and over 20 species of yeasts were reported to occur in artisanal or industrial sourdough fermentation (2, 3). Convergent composition of sourdough microbiota has previously been reported for Type I sourdoughs, which are propagated for use as leavening agent and contain *Kazachstania humilis* in association with *Fructilactobacillus sanfranciscensis* and *Companilactobacillus, Lactiplantibacillus* or *Levilactobacillus* as variable or minor components, and for Type II sourdoughs, which are propagated in industrial bakeries for use as bread improver and are populated by an combination of *Lactobacillus* and *Limosolactobacillus* species (2, 4). The genera that were observed in the pasta sourdough are thus not uncommon in sourdoughs used for baking but the presence of *Pediococcus, Levilactobacillus* and *Fructilactobacillus* species in roughly equal proportions is unusual. Likewise, the occurrence of *Saccharomyces castelii* in sourdough is unprecedented (21), however, other *Saccharomyces* species commonly occur and the species level identification required confirmation by culture. Heterofermentative lactic acid bacteria produce acetic acid from fermentable carbohydrates and other metabolites with antifungal activity (22, 23). Particularly acetic acid production by heterofermentative lactobacilli selects for acid tolerant yeasts in sourdoughs (24) and is a key reason why sourdough bread products have an extended shelf life compared to non-fermented products (25).

Next, we examined the microbial composition of the non-fermented conventional and sourdough pasta products. Conventional pasta exhibited a higher microbial diversity than sourdough pasta with dominance of DNA from bacteria belonging to *Enterobacteriaceae* (19.1%), *Prevotellaceae* (15.8%) and *Lachnospiraceae* (10.8%). These organisms likely represent wheat endophytes and/or contaminants (26, 27). In contrast, 95% of bacterial bacteria in sourdough pasta originated from *Pediococcus* species and likely originates from bacterial cells that remained intact during extrusion and drying until DNAses were inactivated. Analysis of fungus showed the presence of mainly unclassified fungi with greater diversity in the sourdough product. As our analyses detected the presence of microbial DNA rather than viable microorganisms, their presence does not have consequences for product quality or safety.

To compare the metabolic impacts of each pasta, two double blind cross-over studies were conducted. Objectives of the first study (Study A) was to examine gastric emptying, glycemic and hormone responses following the acute consumption of either conventional or sourdough pasta. Previous studies have shown that sourdough fermentation may alter the rate of gastric emptying and therefore contribute to lower glycemic responses. In this study, paracetamol was administered with pasta to track gastric emptying. Results showed shorter gastric emptying with sourdough at 30 and 45 min, and an overall (AUC) difference between the two treatments. The increased rate of gastric emptying at these two time points likely reflects improved starch and protein digestibility. These results are in agreement with Rizzello et al. (28) who tracked gastric emptying of various breads through ultrasound antral area measurements. In that study, the rate of half-emptying time (approximately 30 min post-consumption) was shortest in sourdough vs. baker's yeast bread.

Next, we were interested in examining the glycemic index of each pasta product. Lower glycemic index foods that minimize postprandial disturbances in both glucose and insulin are thought to be key in preventing the development and progression of type 2 diabetes and cardiovascular disease (29, 30). The lower glycemic index with fermented sourdough bread in comparison to white bread has been previously reported (6, 11). Indeed, the addition of organic acids, organic salts and vinegar have been reported to affect both gastric emptying and absorption (31–34). The effect of organic acids on the glycemic index was also attributed to altered starch-protein interactions during baking (35); the impact of sourdough fermentation on the glycemic index of bread is enhanced by the presence of dietary fiber (36). In Study A, results demonstrated no overall differences in glucose or insulin responses between the two pasta treatments. In addition to glucose and insulin, we also examined the influence of the sourdough pasta on gut hormone and incretin responses. In our study, there were no change in C-peptide, GIP, GLP-1, glucagon and PYY following pasta consumption. Of note, the impact of sourdough fermentation on the glycemic index of wheat bread has not been observed in all studies or individuals and appears to be dependent on the individual composition of the intestinal microbiota (11, 37). In addition, differences in the processing of bread, where starch is gelatinized with a limited amount of water during baking, and the processing of pasta, where starch is gelatinized in an excess of water during cooking, may impact starch structure and hence the glycemic index of the products.

To further examine the chronic impacts of sourdough pasta, we undertook a second trial wherein subjects consumed either conventional or sourdough pasta in a cross-over design separated by at least 2 weeks. Subjects consumed 150 g of pasta per day for five consecutive days. While the method of preparation was controlled, the timing (e.g., lunch or dinner consumption) and choice of additives (e.g., pasta sauce) and other foods were not. Employing a standard OGTT to study glycemic responses, we show that pasta had no impact on this parameter. In line with our findings, a crossover trial performed on 20 adults that compared the treatment effects of a week-long consumption of white bread to that of sourdough bread, found that there was no significant difference in glucose response between the two treatment groups (38). It is possible that 1week is not a sufficient time frame to see the expected changes in insulin sensitivity.

Because fermented foods and the gut microbiota are a growing area of interest (39), fecal samples were collected at baseline and following the consumption of each pasta. Samples were assessed for fecal SCFA as well as bacterial and fungal composition. SCFA are major class of microbial metabolites with known beneficial effects on the regulation of glucose and lipid metabolism (40), as well as immune and inflammatory responses (41). Although trending, there was no change in the concentration of individual SCFA with pasta treatments. However, there was a significant decrease in the total concentration of fecal SCFA after chronic consumption of sourdough pasta as compared to baseline values. This data may indicate either reduced production or greater utilization of produced organic acids after chronic sourdough pasta consumption.

Lastly, we examined the impact of chronic pasta consumption on the fecal microbiota. Results showed no change in bacteria or fungi. At the phylum, genus or species levels. However, we observed that sourdough pasta consumption decreased fungal alpha diversity. Fungi are an integral part of the gut microbial environment, but their functional and ecological roles in the mammalian gut microbiomes are not well understood (42). The reasons for this reduction with pasta consumption are not known, however the relationships between host health with bacteria-fungi and host-fungi interactions are becoming more prominent. This microbial niche occupied by fungi is present in healthy individuals and is known to influence the immune system by generating secondary microbial metabolites (43). There is also evidence that obese patients and their metabolic status can be distinguished based on their fungal microbiota. Fungal genus clusters are not only different compared to healthy controls, but can also segregate with adiposity, fasting triglycerides and HDL-cholesterol (44). Sourdough pasta contains no viable fungal cells but any impact of sourdough fermentation on starch digestion or the concentration of fermentable oligosaccharides, disaccharides, monosaccharides and polyols (FODMAP) potentially impacts gut microbiota. Consumption of sourdough bread was previously reported to impact intestinal microbiota, but this is not consistently observed (37, 45). The decreased FODMAP content of sourdough bread (46, 47) is related to alterations in the composition of *in vitro* cultivated intestinal microbiota from healthy volunteers and IBS patients (48).

In conclusion, we found that glucose and insulin responses remain unaltered in overweight individuals after acute and chronic sourdough pasta consumption. We also found that there are no significant changes in gut microbial profile or their associated metabolites after 5 days of dietary intervention. Unexpectedly, consumption of the sourdough product reduced fecal fungal alpha diversity. While the relevance of this finding is not known, it does warrant further investigation.

## Data Availability Statement

The datasets generated for this study can be found in NCBI BioProject, NCBI Accession No. PRJNA668386.

## Ethics Statement

The studies involving human participants were reviewed and approved by Conjoint Health Research Ethics Board at the University of Calgary (REB16-2317). The patients/participants provided their written informed consent to participate in this study.

## Author Contributions

JS, PB, and SL designed and developed the research. SS, PB, SM, CB, and JS conducted experiments, collected, and analyzed data. SS, JS, and CM wrote the manuscript. MG and WW provided data interpretation and manuscript edits and review. All authors revised the manuscript critically for important intellectual content prior to final approval, approved the final version of this manuscript, agree to be accountable for all aspects of the work in ensuring that questions related to the accuracy or integrity of any part of the work are appropriately investigated and resolved, and all persons designated as authors qualify for authorship, and all those who qualify for authorship are listed.

## Conflict of Interest

SL owns and operates Kaslo Sourdough and provided the product for this project. The remaining authors declare that the research was conducted in the absence of any commercial or financial relationships that could be construed as a potential conflict of interest.
