# Supplementary material for: Metabolic and Gut Microbiota Responses to Sourdough Pasta Consumption in Overweight and Obese Adults
Source: Front Nutr. 2020 Dec 23;7:615003. doi: 10.3389/fnut.2020.615003 (PMC7785823; doi:10.3389/fnut.2020.615003)
Supplement: Supplementary file 1 [file Data_Sheet_1.PDF]

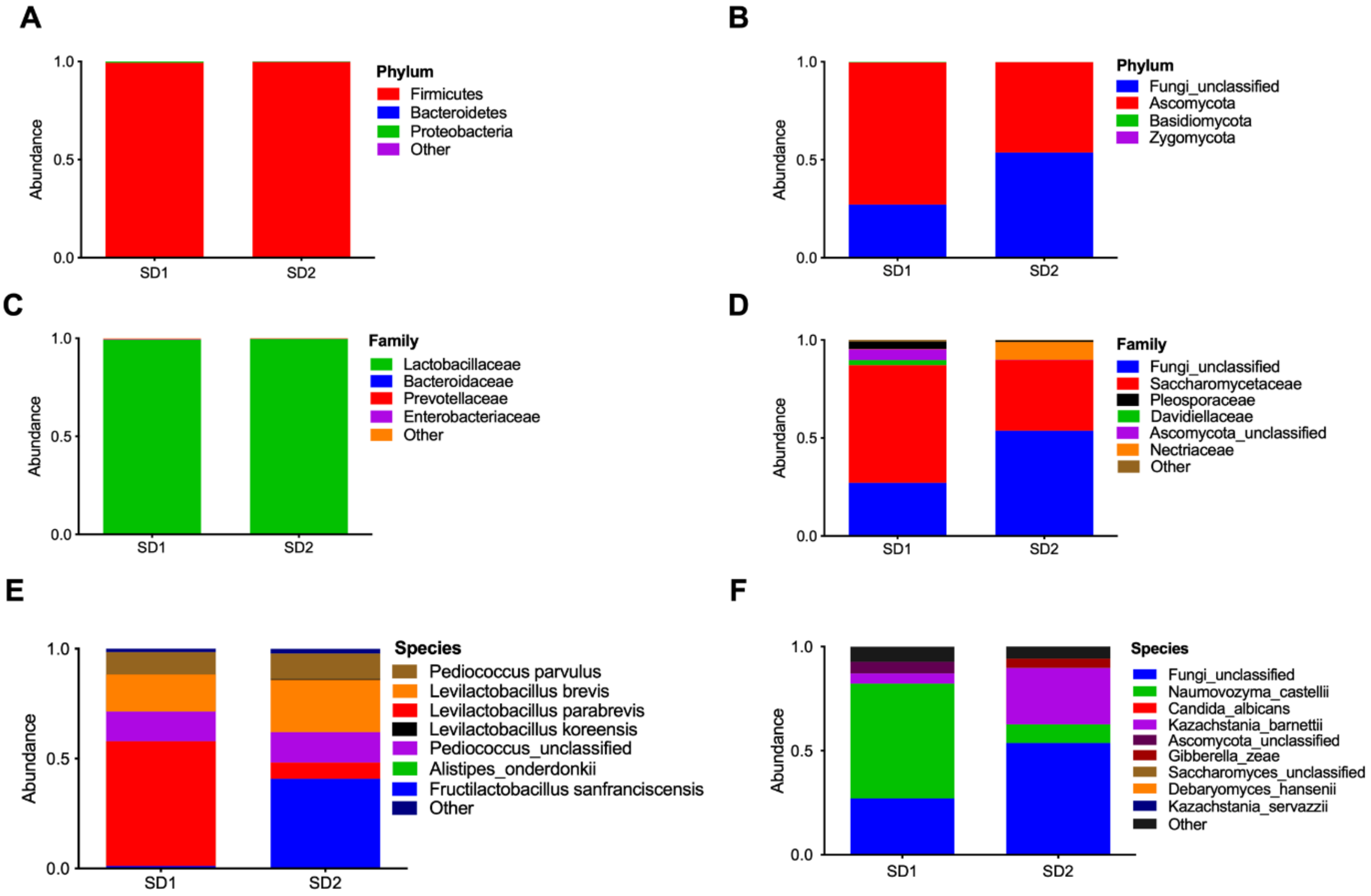

Figure S1. Analysis of bacterial and fungal composition of the sourdough cultures (SD1, mother) and (SD2, mature) at the phylum, family and species levels. A: bacteria phylum, B: fungi phylum, C: bacteria family, D: fungi family, E: bacteria species, F: fungi species.

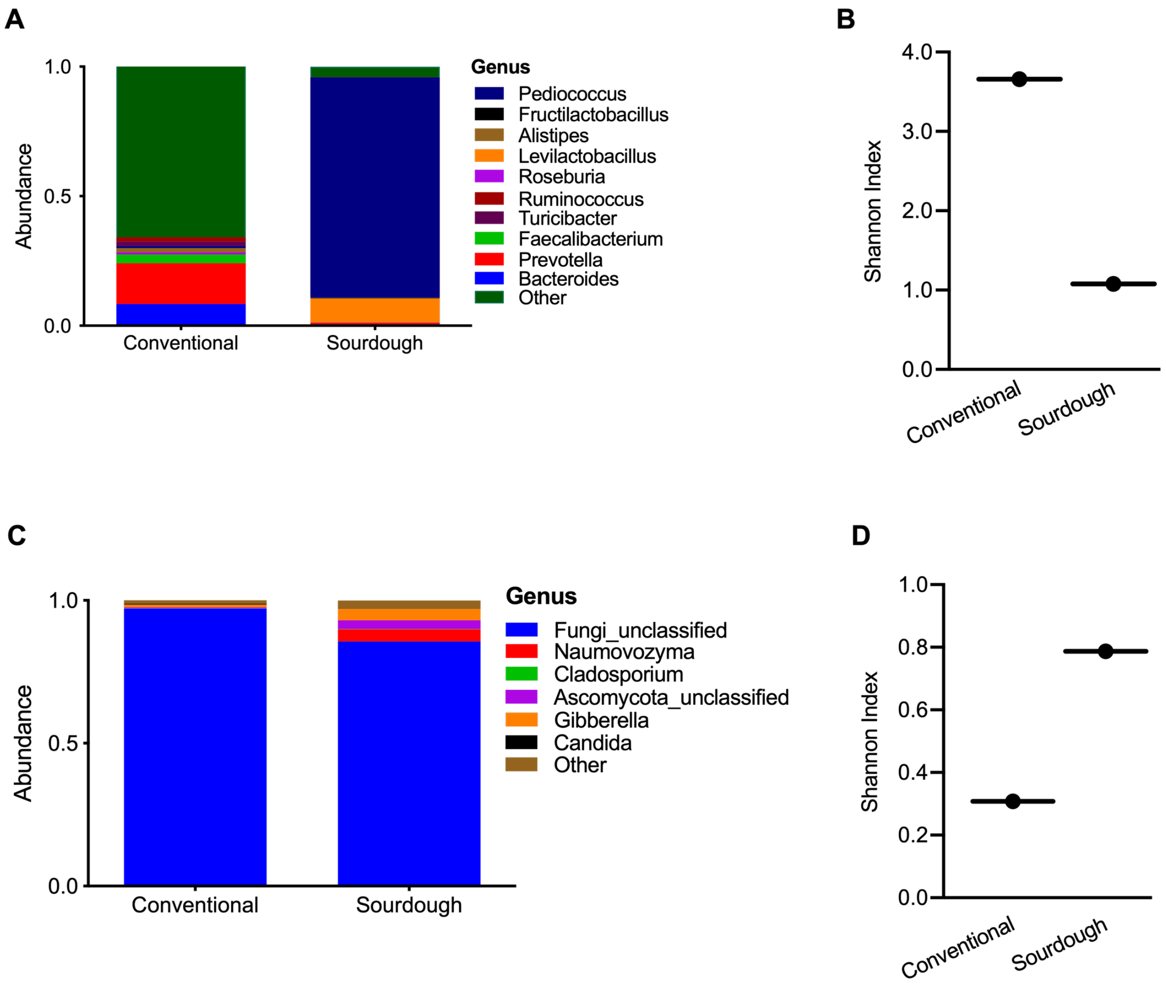

Figure S2. Bacterial and fungal composition of non-fermented conventional and fermented (sourdough) pasta. A) Bacterial genus abundance and B) alpha diversity in conventional vs. sourdough pasta, C) Fungal genus abundance and D) alpha diversity in conventional vs. sourdough pasta

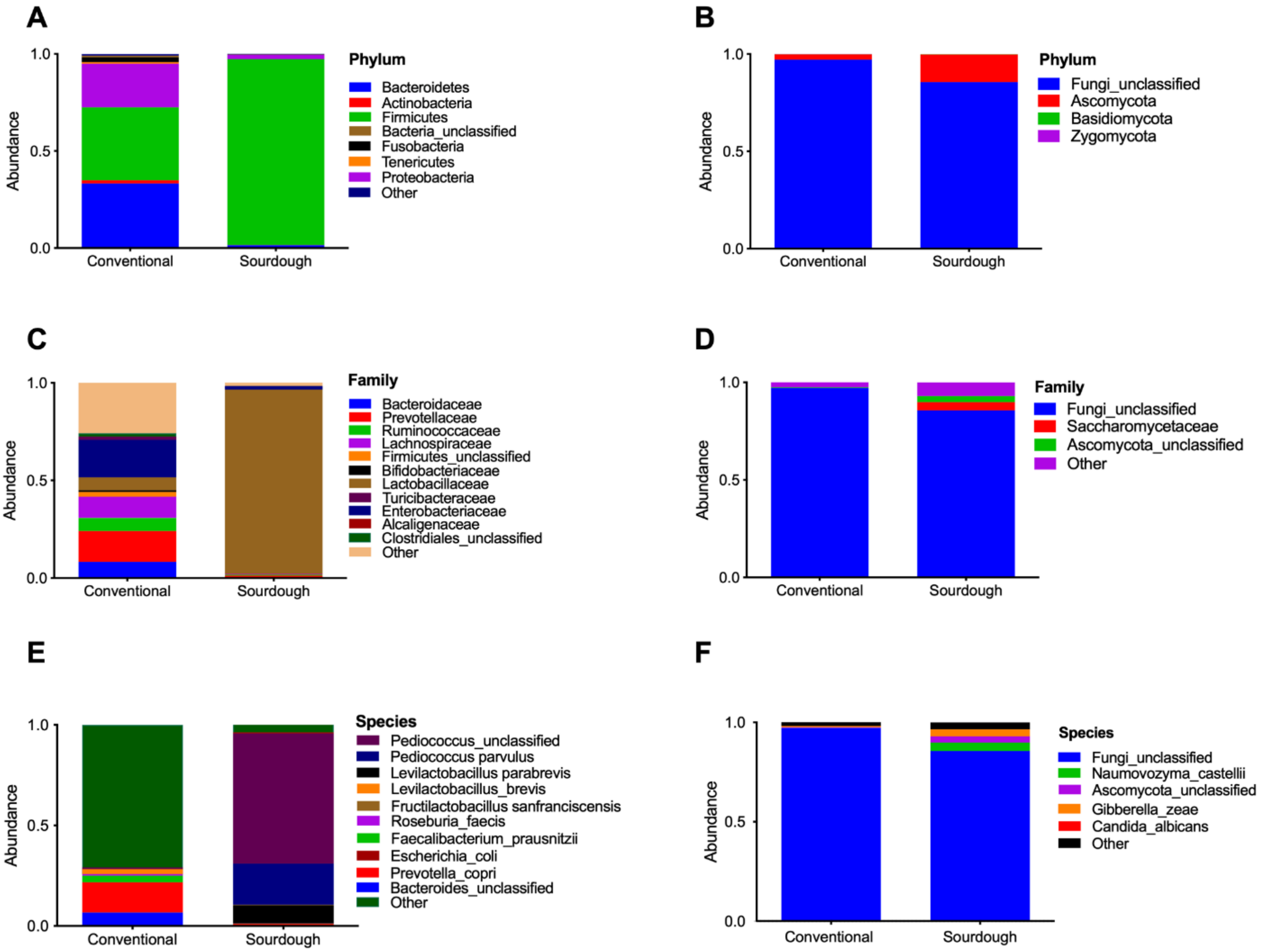

Figure S3. Analysis of bacterial and fungal composition of the conventional and sourdough pasta at the phylum, family and species levels. A: bacteria phylum, B: fungi phylum, C: bacteria family, D: fungi family, E: bacteria species, F: fungi species.

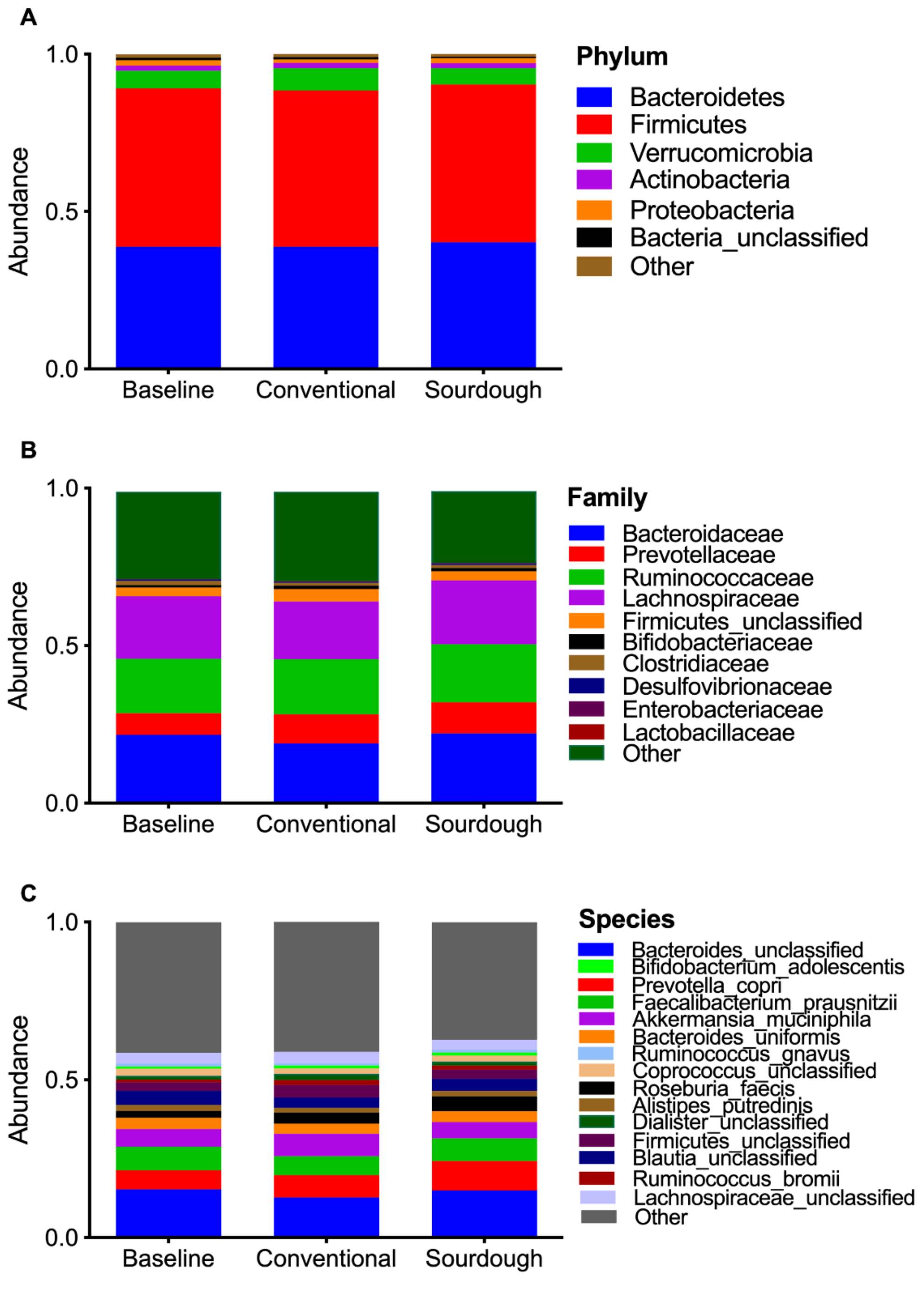

Figure S4. Analysis of bacterial composition of the study participants at the phylum, family and spices levels. A: bacteria phylum, B: bacteria family, C: bacteria species.

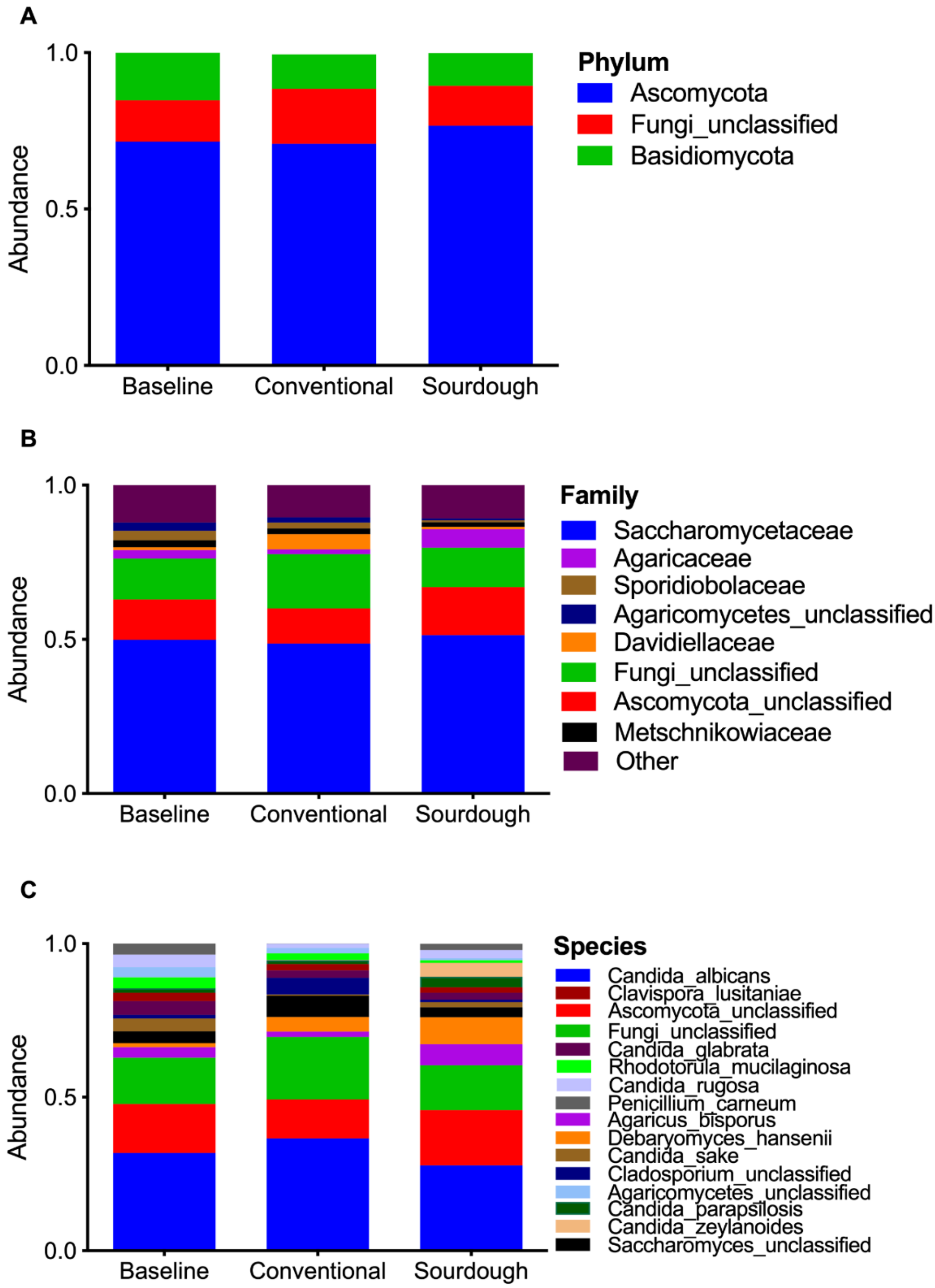

Figure S5. Analysis of fungal composition of the study participants at the phylum, family and spices levels. A: fungi phylum, B: fungi family, C: fungi species.
